# Postpartum development of metabolic dysfunction-associated steatotic liver disease in a lean mouse model of gestational diabetes mellitus

**DOI:** 10.1038/s41598-024-65239-2

**Published:** 2024-06-25

**Authors:** K. Hribar, D. Eichhorn, L. Bongiovanni, M. H. Koster, N. J. Kloosterhuis, A. de Bruin, M. H. Oosterveer, J. K. Kruit, E. M. van der Beek

**Affiliations:** 1 Department of Pediatrics, University of Groningen, University Medical Centre Groningen, Groningen, The Netherlands; 2 Department of Biomolecular Health Sciences, Faculty of Veterinary Medicine, Utrecht University, Utrecht, The Netherlands; 3 Faculty of Veterinary Medicine, University of Teramo, Teramo, Italy; 4 The Central Animal Facility, University of Groningen, University Medical Centre Groningen, Groningen, The Netherlands; 5 Present Address: Nestlé Institute of Health Sciences, Nestlé Research, Lausanne, Switzerland; 6 Department of Laboratory Medicine, University of Groningen, University Medical Centre Groningen, Groningen, The Netherlands

**Keywords:** Gestational diabetes mellitus, Impaired insulin secretion, Metabolic dysfunction-associated steatotic liver disease, Postpartum outcomes, Preclinical model, Endocrine system and metabolic diseases, Non-alcoholic fatty liver disease

## Abstract

Gestational diabetes mellitus (GDM) is associated with increased postpartum risk for metabolic dysfunction-associated steatotic liver disease (MASLD). GDM-related MASLD predisposes to advanced liver disease, necessitating a better understanding of its development in GDM. This preclinical study evaluated the MASLD development in a lean GDM mouse model with impaired insulin secretion capacity. Lean GDM was induced by short-term 60% high-fat diet and low-dose streptozotocin injections (60 mg/kg for 3 days) before mating in C57BL/6N mice. The control dams received only high-fat diet or low-fat diet. Glucose homeostasis was assessed during pregnancy and postpartum, whereas MASLD was assessed on postpartum day 30 (PP30). GDM dams exhibited a transient hyperglycemic phenotype during pregnancy, with hyperglycaemia reappearing after lactation. Lower insulin levels and impaired glucose-induced insulin response were observed in GDM mice during pregnancy and postpartum. At PP30, GDM dams displayed higher hepatic triglyceride content compared controls, along with increased MAS (MASLD) activity scores, indicating lipid accumulation, inflammation, and cell turnover indices. Additionally, at PP30, GDM dams showed elevated plasma liver injury markers. Given the absence of obesity in this double-hit GDM model, the results clearly indicate that impaired insulin secretion driven pregnancy hyperglycaemia has a distinct contribution to the development of postpartum MASLD.

## Introduction

Gestational diabetes mellitus (GDM), defined as diabetes first diagnosed in the second or third trimester of pregnancy^1^, is rapidly increasing worldwide, occurring in approximately 16% of all pregnancies^2^. GDM is recognized as a risk factor for the development of metabolic dysfunction-associated steatotic liver disease (MASLD)^3^, previously known as non-alcoholic fatty liver disease (NAFLD), the most common liver disorder in the Western world^4^. Although premenopausal women have a lower prevalence of MASLD compared to men, a history of GDM increases the risk for MASLD by 2.4 fold^5^. MASLD is a progressive condition that ranges from hepatic steatosis to metabolic dysfunction-associated steatohepatitis (MASH), which is characterized by hepatic inflammation, hepatocyte damage, and fibrosis. MASH is the fastest growing cause for liver transplantation among women due to end-stage liver disease and hepatocellular carcinoma^6–8^. Lifestyle changes, including weight loss and physical activity, are effective in the treatment of MASLD^9^, however, long-term adherence is low, and drug-based treatment options for MASH are scarce^10,11^. Therefore, it is important to identify at-risk individuals prior to the development of advanced liver disease.

GDM is a heterogeneous disease, with subtypes identified based on the degree of insulin resistance. Most studies have focused on overweight and obese women with GDM characterized predominately by increased insulin resistance^12^; however, a significant proportion of women with GDM are lean and insulin-sensitive, characterized predominately by a decreased insulin secretion capacity^13–16^. Although the aetiology of GDM subtypes differs, both subtypes have an increased risk of developing postpartum impaired glucose tolerance^17^. Since insulin resistance is a critical pathophysiological factor in MASLD^18^, it is questionable whether insulin sensitive GDM subtypes show similar MASLD risk. Clinical data on postpartum MASLD development in different GDM subtypes is lacking.

Recently, we established a lean GDM mouse model that combines high fat feeding and limiting beta-cell function and expantion by streptozotocin (STZ) treatment^19,20^ that mimics the phenotype of lean insulin sensitive GDM. Using this double-hit lean GDM mouse model, we aimed to determine whether the lean, insulin sensitive GDM subtype poses an increased risk for the postpartum development of MASLD. To this end, we studied glucose homeostasis during pregnancy and lactation up to postpartum day 30 (PP30) and MASLD development at PP30.

## Materials and methods

### Animals

Animal procedures were performed in compliance with EU legislation (Directive 2010/63/EU). The ethical license was approved by the Dutch Central Committee on Animal Testing and the study protocol was approved by the Central Authority for Scientific Procedures on Animals of the University of Groningen. The study is reported in accordance with ARRIVE guidelines. All institutional and national guidelines for the care and use of laboratory animals were followed in this study. In total 74, 9-week-old nulliparous female and 35 8-week-old nulliparous male C57BL/6NTac mice were purchased from Taconic, Denmark. Mice were housed in individually ventilated cages under a 12h light–dark cycle (7 AM-7 PM). Females were pair-housed until gestation and single housed afterwards and fed a 10E% low fat diet (LF; D12450Ji, Research Diets) or 60E% high fat diet (HF; D12492i, Research Diets). Males were housed individually and fed chow diet (AB diet). Body weight (BW) was monitored weekly. The experimenters were not blinded, except for the pathologist who performed the histopathological analysis.

### Experimental setup

**Figure 1:**
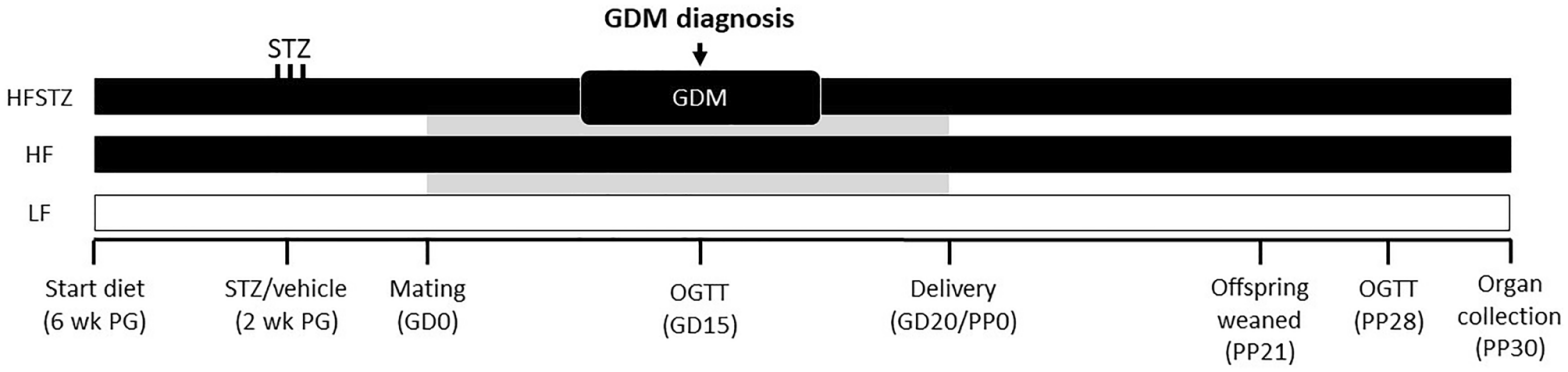
Schematic overview of the GDM postpartum follow-up experimental design. Mice were fed an HF or LF diet starting six weeks prior to gestation (PG). After 4 weeks of feeding, the mice were administered STZ or vehicle i.p. for three consecutive days. On GD15, GDM was confirmed by an OGTT. The litter size was determined and standardized to 3F + 2M at PP2. Postpartum recovery was evaluated using random blood glucose measurements at PP15. Offspring were removed at PP21. Glucose homeostasis was evaluated using the OGTT at PP28. The dams were terminated on PP30.

Upon arrival, the females were assigned to one of three groups using a computer-generated sequence (Research Randomizer, randomizer.org). The treatment groups included LF + vehicle (LF), HF + vehicle (HF), and HF + STZ (HFSTZ). Briefly, after four weeks of feeding, mice received either 60 mg/kg streptozotocin (STZ) (S0130, Sigma-Aldrich) (HFSTZ group) or vehicle (LF, HF groups) for three consecutive days, as described previously^19,20^. Twelve days after STZ or vehicle injection, vaginal smears were taken at 3 PM daily to assess the oestrus cycle. Mice were bred as previously described^19^. The litters were standardized to three females and two males at postpartum day 2 (PP2), offspring was weaned at PP21 and used in a separate experiment. The effect of maternal GDM on the postpartum development of metabolic dysfunction-associated steatotic liver disease was studied using the PP30 time point. The experimental overview is presented in Fig. 1. Dams were terminated in random order by cardiac puncture under isoflurane anaesthesia. Tissues were excised, snap-frozen, or fixed in 4% (w/v) formaldehyde in PBS.

### Hyperglycaemia detection and cut-offs for diabetes diagnosis

To monitor glycaemic changes indicating diabetes development, random blood glucose (RBG) levels under non-fasting conditions were analysed in blood samples collected from a tail cut using a handheld glucose meter (Accu-Chek Performa, Roche). RBG was measured in a random order between 9 AM-10 AM prior to STZ injection, at GD0/GD7/GD14/GD18, PP8/PP15/PP23, and PP30. Based on phenotype, HFSTZ-treated dams were subdivided into pre-gestational diabetes mellitus (PDM) and GDM groups. HFSTZ mice that developed hyperglycaemia prior to pregnancy (defined as RBG > 12 mmol/l on GD0) were classified as PDM and removed from the study. Dams with RBG < 12 mmol/l on GD0 were classified as normoglycemic, mated and screened for GDM using a GD15 OGTT.

### Oral glucose tolerance test (OGTT)

The OGTT was performed on GD15, roughly corresponding to the moment of clinical GDM diagnosis, and at PP28, to assess postpartum T2DM development. Females were fasted for 6h (6 AM-12 PM, lightphase) in their home cage, followed by fasting blood glucose measurement and collection of a small blood sample from the tail on filter paper (Satorius stedim TFN, 180 g/m2). Glucose tolerance was subsequently assessed using OGTT. D-Glucose (1 g/kg BW in 200 g/L solution) was administered by oral gavage. Blood glucose (BG) measurements were performed at 0, 5, 10, 20, 30, 45, 60, 90 and 120min. Additionally, blood spots to quantify insulin levels were obtained at six time points (see below). HFSTZ-treated dams that were normoglycemic at GD0 were diagnosed with GDM if the 2h OGTT BG level was > 12 mmol/l.

### Insulin levels, HOMA-IR, and Matsuda index

Blood samples for insulin measurement were collected on filter paper by tail bleeding for random insulin determination on GD0, GD18, PP15, PP30, and during OGTT at 0, 5, 10, 30, 60 and 120 min. Insulin levels were measured using ELISA (Crystal Chem Cat. #90060) as previously described^19^. Insulin levels on the day of termination (PP30) were quantified by ELISA (Crystal Chem) using 5 µL of plasma. To correct for differences in sample volumes between the blood spots and plasma samples, the concentrations derived from the blood spots were multiplied by 1.28^21^. HOMA-IR was calculated as previously described^22^. The Matsuda index was calculated as previously^23^.

### Biochemical liver analysis

Hepatic lipids were extracted from 15% (w/v) liver homogenates in PBS, according to the method described by Bligh and Dyer^24,25^. Hepatic TGs (Roche), free (DiaSys), and total (Roche) cholesterol levels were analysed using commercially available kits. The concentration of cholesteryl esters was calculated as the difference between the total and free cholesterol concentrations. Hepatic phospholipids were analysed as described previously^26^.

### Histological liver analysis

For microscopic examination, the tissues were fixed in 4% (w/v) formaldehyde in PBS, embedded in paraffin, sectioned at 4 µm, and stained with Hematoxylin and Eosin (H&E) and Periodic Acid Schiff (PAS). Representative photomicrographs per liver were taken at 10 × and 40 × using Aperio ImageScope 12.1, after scanning the stained sections with a Hamamatsu NanoZoomer (Hamamatsu Photonics). Histopathological scoring of MASLD lesions, apoptotic cells and mitotic figures in H&E-stained liver sections was performed by a board-certified veterinary pathologist as previously described^27^.

### Plasma analysis

Plasma TGs and non-esterified fatty acids (NEFAs) were measured using the same kits (Roche Diagnostics). Plasma leptin levels were analysed using a Mouse Leptin ELISA kit (Cat. #90030, Crystal Chem), according to the manufacturer’s protocol. Plasma alanine aminotransferase (ALT) and aspartate transaminase (AST) levels were analysed using a Cobas 6000 analyser with standard reagents (Roche Diagnostics).

### Statistics

Data are presented as mean ± SD, using the dam as the experimental unit. The power calculation and final number of animals included are described in the supplements (Supplemental Table S1). Normality was evaluated and one-way ANOVA and Tukey's multiple comparison test was used for normally distributed data or Kruskal–Wallis test and Dunn's multiple comparisons test for non-normally distributed data. For time-course graphs, two-way ANOVA followed by Tukey's multiple comparison test was used. Correlations within the GDM group were assessed using Pearson’s or Spearman’s correlation coefficients. All statistical analyses were performed using GraphPad Prism 10.1.2. Two-sided p < 0.05 was considered statistically significant.

## Results

### Despite high fat feeding, GDM mice display a lean phenotype postpartum

**Figure 2:**
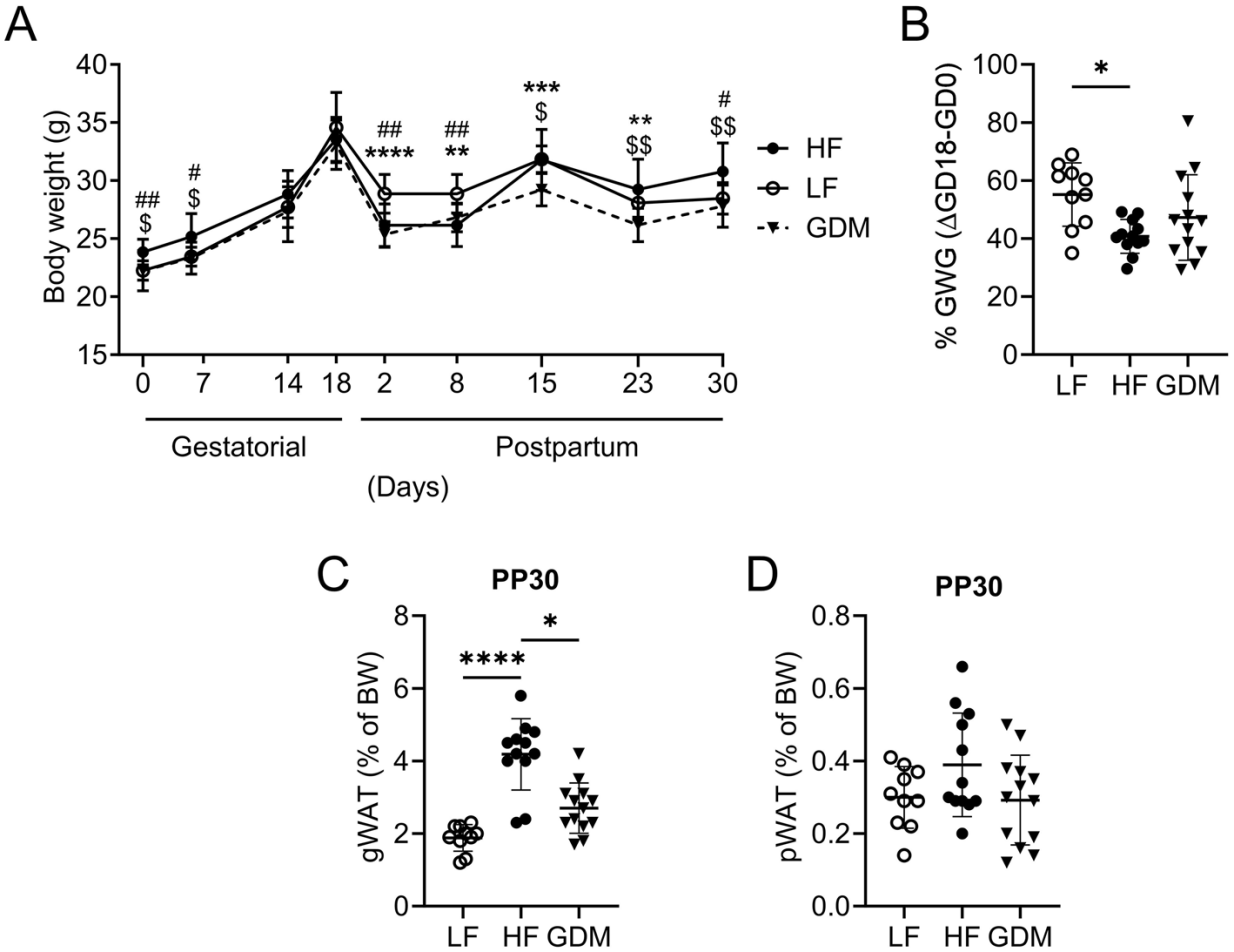
GDM dams remain lean despite HF feeding. (**A**) Body weight of dams through the gestational and postpartum periods. (**B**) Gestational weight gain (%GWG, ∆GD18-GD0). (**C**) Gonadal (gWAT) and (**D**) perirenal (pWAT) tissue fat pads obtained at PP30 are presented as percentages of the body weight. *LF* low-fat diet, *HF* high-fat diet, *GDM* gestational diabetes mellitus, *pWAT* perirenal white adipose tissue, *gWAT* gonadal white adipose tissue, (**A**–**D**) LF: n = 10, HF: n = 12, GDM: n = 13. Data are presented as the mean ± SD. **A:** Two-way ANOVA followed by Tukey’s multiple comparison test. (**B**–**D**) Kruskal–Wallis test followed by Tukey’s multiple comparison test. *GDM vs. LF, ^$^GDM vs. HF, ^#^HF vs. LF. *p < 0.05, **p < 0.01, ***p < 0.001, ****p < 0.0001.

Although six weeks of high fat diet (HF) feeding increased the weight of HF dams before mating (gestational day 0; GD0), the GD0 weight of GDM dams was statistically indistinguishable of that of low fat diet (LF) control dams (Fig. 2A). Gestational weight gain (GWG; ∆GD18-GD0) was lower in GDM and HF dams than in LF dams (Fig. 2B). During lactation (postpartum (PP) day 2 to day 23), the GDM and HF dams were lighter than the LF dams. While the weight of LF dams remained stable after weaning of the pups (PP21), GDM and HF dams regained weight after PP21. At PP30 HF dams showed significantly increased body weight and gonadal adipose tissue compared to GDM and LF dams. Notably, the body weight and fat mass of GDM dams were similar to those of LF controls at PP30 (Fig. 2A,C,D). At PP30, no differences in plasma leptin levels were detected between groups (Table 1). These observations suggest that GDM dams remained lean despite HF feeding.

**Table 1:** Plasma parameters of dams at PP30.

	LF N = 10	HF N = 12	GDM N = 13	LF vs. HF	LF vs. GDM	HF vs. GDM
ALT (U/L)	71.50 ± 18.42	63.75 ± 13.51	155.00 ± 61.61	ns	0.0015	0.0002
AST (U/L)	24.00 ± 6.99	26.67 ± 22.09	81.92 ± 45.26	ns	0.0009	< 0.0001
Cholesterol (mM/L)	2.48 ± 0.39	2.88 ± 0.42	2.76 ± 0.27	0.0462	ns	ns
Triglycerides (mM/L)	0.44 ± 0.17	0.39 ± 0.19	0.41 ± 0.23	ns	ns	ns
NEFAs (mM/L)	0.45 ± 0.11	0.70 ± 0.24	0.70 ± 0.27	0.0193	0.0121	ns
Leptin (ng/ml)	19.75 ± 9.20	25.96 ± 10.28	18.21 ± 7.63	ns	ns	ns

Data are presented as the mean ± SD. Statistical analysis was performed using Kruskal–Wallis test, followed by Dunn’s multiple comparison test. P values are presented as group comparisons.

*LF* low-fat diet, *HF* high-fat diet, *GDM* gestational diabetes mellitus, *AST* plasma aspartate transaminase, *ALT* plasma alanine aminotransferase.

### Lean GDM mice show impaired glucose tolerance, perturbed insulin response and insulin resistance during pregnancy and postpartum

**Figure 3:**
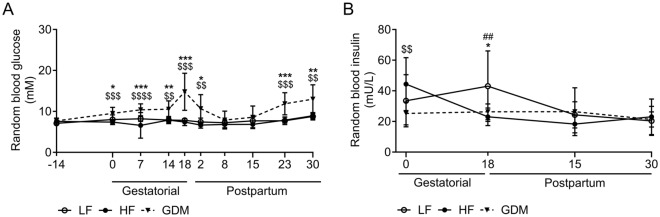
Transient hyperglycaemia and reduced insulin concentrations in GDM dams. (**A**) Non-fasted random blood glucose concentrations between the moment prior to streptozotocin (STZ) injections (GD -14) and PP30. (**B**) Non-fasted random insulin concentrations throughout pregnancy, lactation, and the postpartum period. LF: Low-fat diet, HF: high-fat diet, GDM: gestational diabetes mellitus, (**A**,**B**) LF: n = 10, HF: n = 12, GDM: n = 13. Data are presented as the mean ± SD. Mixed effects 2-way ANOVA followed by Tukey’s multiple comparison test. *GDM vs. LF, ^$^GDM vs. HF, ^#^HF vs. LF. *p < 0.05, **p < 0.01, ***p < 0.001, ****p < 0.0001.

GDM dams exhibit a transient hyperglycaemic phenotype. During pregnancy, GDM dams peak in random blood glucose concentrations at the end of GD18, followed by postpartum normalization of RBG levels during lactation (PP8/PP15, Fig. 3A). Interestingly, hyperglycaemia reappeared after lactation (PP23/30; Fig. 3A). While GDM dams displayed consistently higher glucose levels during gestation, their random blood insulin concentrations remained low compared to those of the HF and LF dams. Gestation decreased insulin levels in HF dams, whereas LF dams showed stable insulin levels with a slight increase during pregnancy. Insulin concentrations were similar between groups at lactation (PP15) and during the postpartum period (PP30) (Fig. 3B). Individual values for RBG and insulin are shown in Supplementary Table S2.

**Figure 4:**
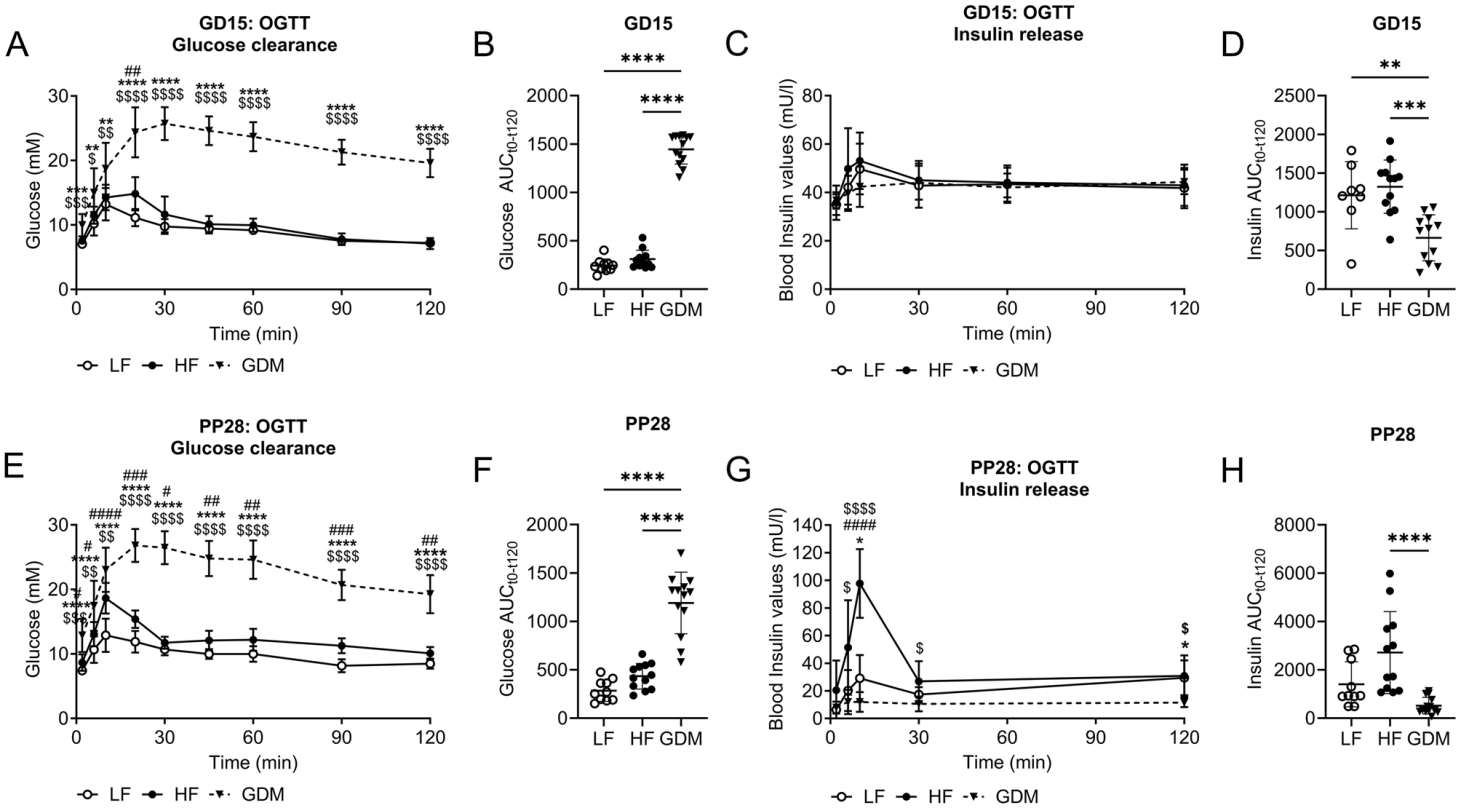
Glucose tolerance and insulin response during OGTT in pregnancy and postpartum. (**A**) Blood glucose concentration throughout the OGTT at GD15. (**B**) GD15 OGTT Glucose AUC corrected for t0. (**C**) Blood insulin concentration throughout the OGTT at GD15. (**D**) GD15 OGTT Insulin AUC corrected for t0. (**E**) Blood glucose concentrations throughout the OGTT at PP28. (**F**) PP28 OGTT glucose AUC corrected for t0. (**G**) Blood insulin concentration throughout the OGTT at PP28. (**H**) PP28 OGTT Insulin AUC was corrected for t0. (**A**–**H**) LF: n = 10, HF: n = 12, GDM: n = 13. Data are presented as the mean ± SD. (**A**,**C**,**E**,**G**) Mixed effects 2-way ANOVA followed by Tukey’s multiple comparison test. (**B**,**D**,**F**,**H**) Kruskal–Wallis test followed by Tukey’s multiple comparison test. *GDM vs. LF, ^$^GDM vs. HF, ^#^HF vs. LF. *p < 0.05, **p < 0.01, ***p < 0.001, ****p < 0.0001.

In addition to measuring RBG and blood insulin levels, we performed an oral glucose tolerance test (OGTT) on GD15 to confirm GDM diagnosis and evaluate systemic glucose tolerance in the dams. GDM dams showed severely impaired glucose tolerance, as evidenced by the increased blood glucose levels throughout the OGTT (Fig. 4A,B; Suppl. Table S3). HF dams showed a moderate increase in blood glucose levels, which reached statistical significance only 20 min after the glucose bolus compared to LF dams (Fig. 4A,B; Suppl. Table S3). At PP15 GDM dams showed significantly lower insulin levels than the HF and LF dams during the first 30 min of the OGTT (Fig. 4C,D; Suppl. Table S3).

Postlactation glucose management was evaluated using an OGTT at PP28. Fasted blood glucose levels prior to the PP28 OGTT were increased in the GDM group compared to LF and HF dams (Fig. 4E; Suppl. Table S3). Throughout the PP28 OGTT, blood glucose levels in GDM dams were significantly higher than those in LF and HF dams, whereas blood glucose levels in HF dams were significantly increased throughout the OGTT compared to LF controls (Fig. 4E,F; Suppl. Table S3). GDM dams showed impaired insulin release after the glucose bolus, with significantly lower insulin levels than the LF and HF dams throughout the PP28 OGTT (Fig. 4G,H; Suppl. Table S3). The OGTT insulin response of HF dams was significantly higher than that of GDM and LF dams at PP28, suggesting that postpartum insulin resistance developed as a result of continued exposure to HF (Fig. 4G,H; Suppl. Table S3).

**Figure 5:**
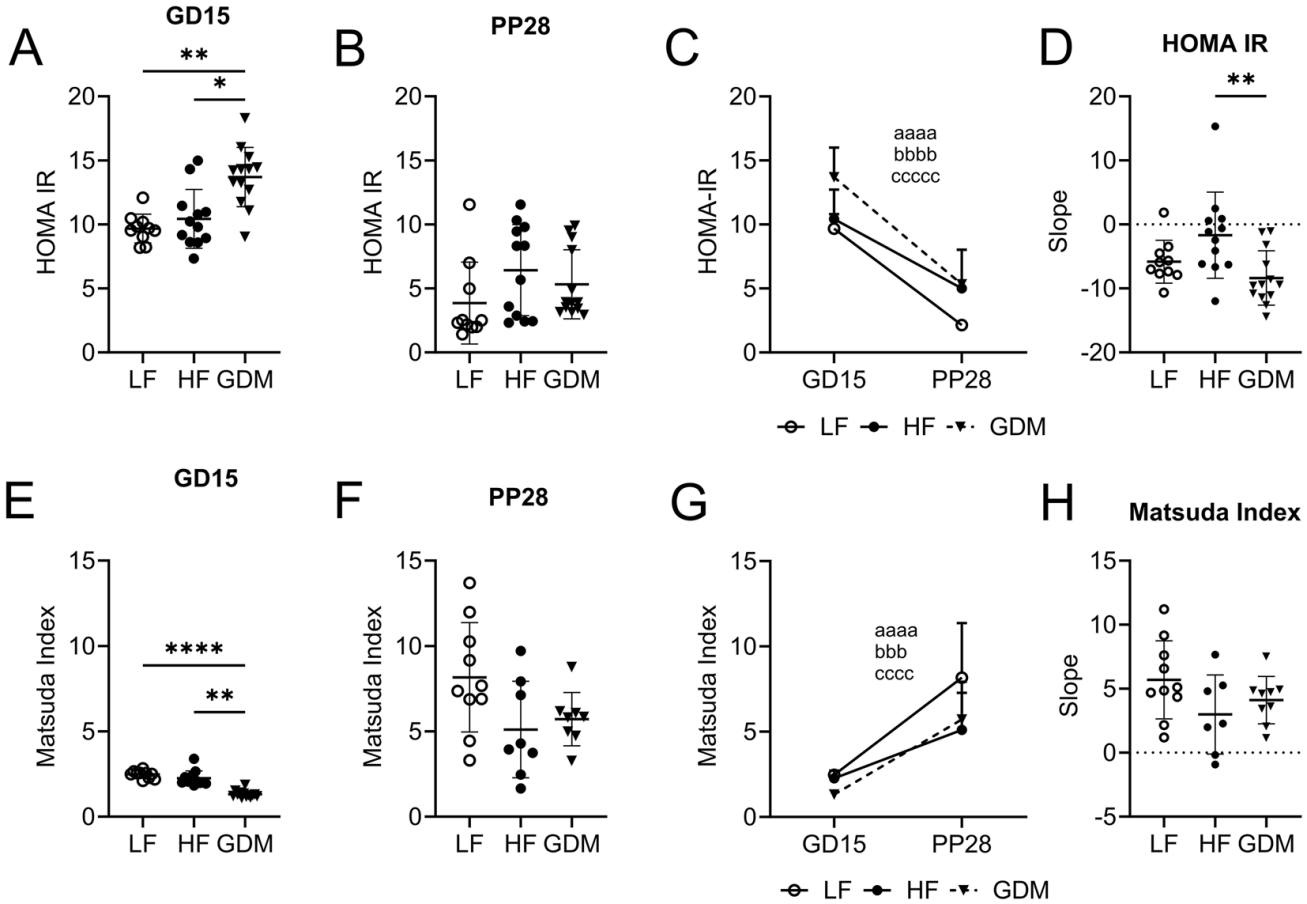
Changes in insulin sensitivity during gestation and postpartum. (**A**–**C**) HOMA-IR derived from the GD15 and PP28 fasting glucose and insulin concentrations. (**E**–**G**) Matsuda Index derived from the GD15 and PP28 fasting glucose and insulin concentrations. Slope of GD15-PP28 of HOMA-IR (**D**) and Matsuda Index (**H**). (**A**–**H**) LF: n = 10, HF: n = 12, GDM: n = 13. Data are presented as mean ± SD. Kruskal–Wallis test followed by Tukey’s multiple comparison test. ^a^GDM vs. GDM, ^b^HF vs. HF, ^c^LF vs. LF. *p < 0.05, **p < 0.01, ***p < 0.001, ****p < 0.0001.

On GD15, GDM dams showed decreased insulin sensitivity, assessed by HOMA-IR and Matsuda indices, compared to HF and LF dams (Fig. 5A,B,E,F; Suppl. Table S3). Pregnancy normally reduces insulin sensitivity in dams to ensure adequate glucose supply to the foetus. Insulin sensitivity was improved after gestation in all dams at PP28 (Fig. 5C,D,G,H; Suppl. Table S3). HF dams showed similar insulin sensitivity to LF animals during gestation but failed to improve to the same level as LF and GDM dams at PP28 (Fig. 5D). Plasma NEFAs, often associated with insulin resistance, were similarly increased in GDM and HF dams vs LF controls (Table 1). In summary, the collected data suggests that post-gestation, HF dams normalize the insulin sensitivity to a lesser extent than LF or GDM dams. Post gestation, GDM dams do not show significant differences in insulin sensitivity compared to LF dams. Instead, GDM dams are characterized by lower glucose-induced insulin secretion.

### Lean GDM leads to the development of postpartum MASLD

**Figure 6:**
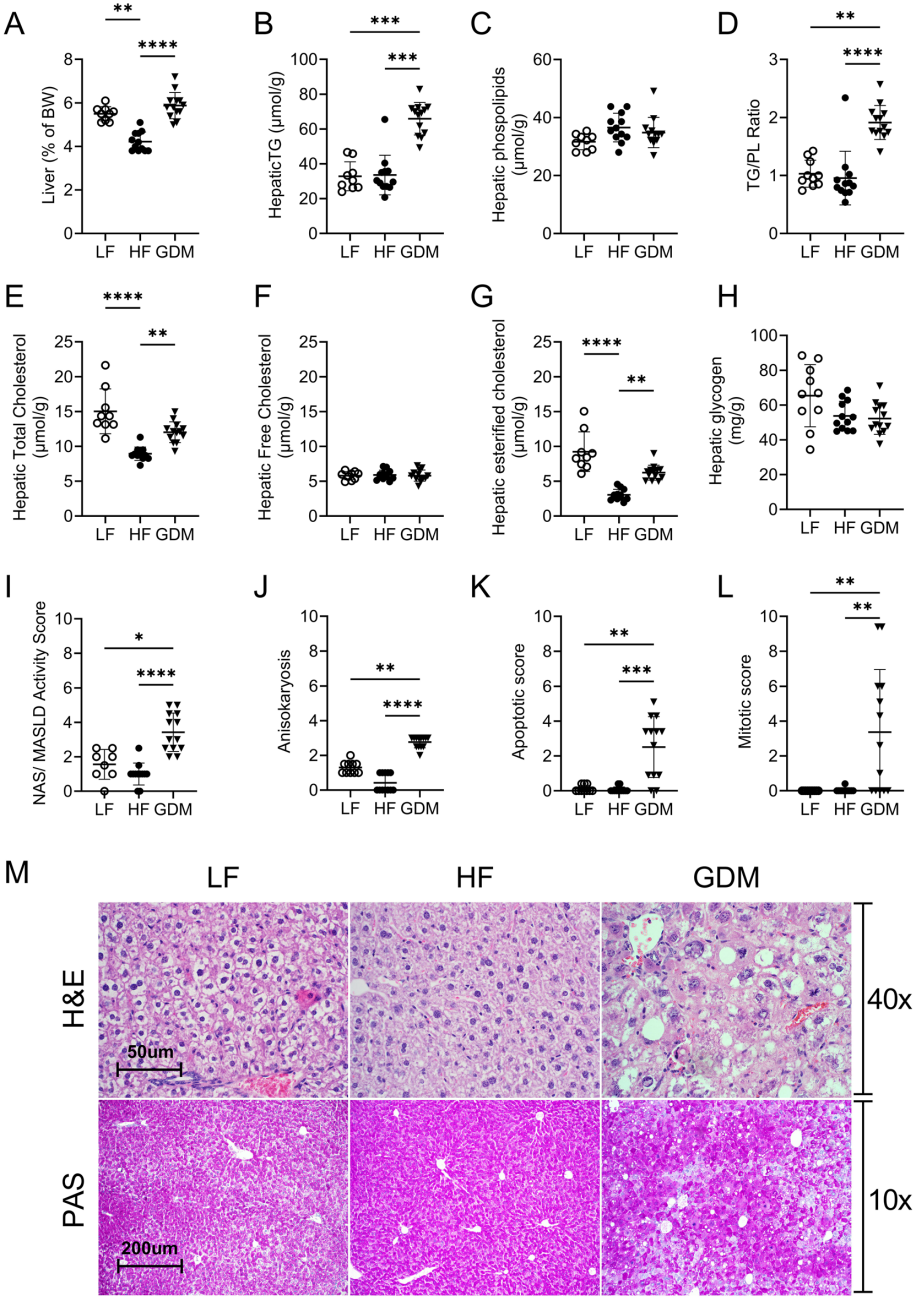
PP30 liver weight, lipid content and liver morphology. (**A**) Liver weight presented as percentage of body weight. (**B**) Hepatic triglycerides. (**C**) Hepatic phospholipids. (**D**) Hepatic TG/PLs ratios. (**E**) Hepatic total cholesterol. (**F**) Hepatic free cholesterol. (**G**) Hepatic esterified cholesterol contents. (**H**) Hepatic glycogen. (**I**) MASLD activity score (MAS), compromising of lobular inflammation, steatosis, grade, and ballooning. (**J**) Anisokaryosis derived from the pathological assessment of liver H&E slides. (**K**) Apoptotic scores derived from pathological assessment of H&E-stained liver tissue sections. (**L**) Mitotic figure scores derived from pathological assessment of H&E-stained liver tissue sections. (**M**) Representative images of H&E- and PAS-stained liver sections. *LF* low-fat diet, *HF* high-fat diet, *GDM* gestational diabetes mellitus, LF: n = 10, HF: n = 12, GDM: n = 13. Data are presented as the mean ± SD. (**A**–**J**,**L**–**M**) Kruskal–Wallis test followed by Tukey’s multiple comparison test. *p < 0.05, **p < 0.01, ***p < 0.001, ****p < 0.0001.

To determine whether GDM dams develop postpartum MASLD, livers were analyzed at PP30. The relative liver weights of GDM dams did not differ from those of LF dams, but were increased compared to HF dams (Fig. 6A). GDM dams showed significantly higher liver triglyceride (TG) content as compared to HF and LF dams (Fig. 6B; Suppl. Table S4), indicating liver steatosis in GDM dams. Free Cholesterol didn’t differ between groups (Fig. 6F; Suppl. Table S4). Unexpectedly, total hepatic cholesterol and cholesterol ester contents were also markedly higher in the GDM group than in the HF group, but not in the LF dams (Fig. 6E,G; Suppl. Table S4). The TG/phopholipids (PL) ratio, a marker of lipid droplet size, was significantly increased in the liver of GDM dams compared to that in HF and LF dams (Fig. 6C,D; Suppl. Table S4).

Consistent with the accumulation of hepatic TGs, cholesterol esters, and an increased TG/PL ratio, histopathological analysis showed increased MASLD activity scores (MAS) in livers from GDM compared to HF and LF dams (Fig. 6I; Suppl. Table S4). The MASLD activity score (MAS) was derived from the pathological assessment of liver hematoxylin and eosin (H&E) slides and included lobular inflammation, steatosis grade, and ballooning. Histological analysis of the liver revealed that the GDM dams showed hepatocyte hypertrophy with hepatocytes that were nearly twice the size (half as many per 20 × field [0.95 mm^2^] as compared with other groups; Fig. 6M; Suppl. Table S4). Hypertrophy was associated with panzonal accumulation of lipids (on average 33–66% of hepatocytes). There were no significant differences in glycogen accumulation (Fig. 6H,M; Suppl. Table S4). Anisokaryosis score (differences in nuclear sizes) were also higher in GDM compared to HF and LF dams (Fig. 6J; Suppl. Table S4). In addition, we found an increase in the number of apoptotic cells (Fig. 6K; Suppl. Table S4) and mitotic figures (Fig. 6L; Suppl. Table S4) in the livers of GDM dams compared to HF and LF dams.

Finally, MASLD is usually asymptomatic and is screened by measuring aspartate aminotransferase (AST) and alanine aminotransferase (ALT) levels. Only the GDM dams showed an increase in plasma liver injury markers AST and ALT compared to HF and LF dams (Table 1). GDM did not affect plasma lipid levels as plasma TG or cholesterol levels were similar between GDM and HF dams (Table 1). HF dams showed a slight increase in plasma cholesterol as compared to LF dams (Table 1).

## Discussion

Using a previously established mouse model of GDM^19,20^, we show that lean GDM driven by impaired insulin secretion is characterized by a transient hyperglycaemia during pregnancy which reappeared after the lactation period. Despite low insulin levels, our GDM model displayed significantly increased hepatic lipid contents coinciding with increased MASLD activity scores at 30 days postpartum. These findings suggest that not only GDM subtypes driven by insulin resistance, but also GDM subtypes characterized by impaired insulin secretion are at risk to develop postpartum MASLD.

GDM is increasingly recognized as a heterogenous disease^14^. The two main GDM subtypes are distinguished by a predominant defect in insulin sensitivity versus a predominant defect in insulin secretion^14,28,29^. These GDM subtypes have varying clinical characteristics and risks of short-term complications^30,31^. Depending on the population, the prevalence of the insulin-deficient subtype ranges between 20 and 30%^14,30^. Compared to insulin resistant GDM, women with the insulin-deficient GDM subtype have a lower BMI and are by definition more insulin sensitive^14^. Although both GDM subtypes show similar glucose intolerance postpartum^32–34^, the current research landscape lacks a comprehensive exploration of the long-term risk of negative health consequences in the various subtypes of GDM, including postpartum type 2 diabetes mellitus (T2DM) or MASLD development^35,36^. While reduced insulin sensitivity is a key metabolic disturbance linked to MASLD^37,38^, a large retrospective cohort study has underscored that a history of GDM, independent of insulin resistance or diabetes, constitutes an independent risk factor for the development of MASLD^36^.

Our lean GDM mouse model mimics the impaired insulin-secretion-driven GDM observed in humans^14–17,39^. Insulin secretion capacity of beta-cells was limited by administration of a low dose of streptozotocin 2 weeks prior mating. At GD0, random blood glucose levels were moderetaly increased in the GDM group, but not to a level which classifies the model as a diabetes model^19^. A possible limitation of our model is that streptozotocin treatment itself in non-pregnant female mice result in impaired glucose tolerance and decreased beta-cell mass^19^. Pregnancy, however, led to a further impairment of glucose homeostasis characterized by increased random and fasted glucose levels and a profound glucose intolerance combined with reduced insulin levels^19^, demonstrating it is an impaired insulin-secretion-driven GDM model. Insulin sensitivity changes during pregnancy as a result of several changes in hormonal release^40–42^. In the latter half of gestation, there is a notable decline in insulin sensitivity, and this reduction can be particularly pronounced in women with GDM^43^. Our study aligns with this pattern, as all dams exhibited decreased insulin sensitivity during pregnancy compared to the postpartum period. Notably, in GDM dams, insulin sensitivity was significantly decreased compared with LF and HF dams during gestation. Postpartum GDM dams did not exhibit significant differences in insulin sensitivity compared LF and HF dams. Instead, GDM dams are characterized by lower glucose-induced insulin secretion in comparison to their LF and HF counterparts.

During pregnancy, GDM dams peak in random blood glucose levels at the end of gestation, followed by postpartum normalization of random blood glucose levels until the pups are weaned. It has been reported previously that women with GDM who breastfed also showed a reduction in fasting glucose postpartum compared with women who did not breastfeed^44,45^. Lactation may improve glucose metabolism and insulin sensitivity by increasing systemic glucose disposal rates^46^ and increasing FFA flux from adipose tissue to the mammary gland^47^. Indeed, longer duration of lactation is associated with a lower risk of T2DM and a favourable metabolic profile among women with a history of GDM^44,48^. In addition, prevalence of MASLD is lower in women with longer duration of lactation^5^. In our study the protective effect of breastfeeding on glucose levels in GDM dams did not persist beyond the breastfeeding period. The transiently improved hyperglycaemia may also be related to prolactin levels. Prolactin levels will only spike during periods of nipple stimulation through suckling by offspring. As long as the offspring-maintained suckling prolactin levels likely remained elevated. Prolactin is involved in pancreatic structure and function, and prolactin levels have been shown to be related to maternal T2DM risk^48,49^. In our study we were not able to measure prolactin levels, but the relationship between maternal prolactin and the resurgence of hyperglycaemia in women with previous GDM has been confirmed in previous studies^50^.

Notably, GDM dams, but not HF dams, exhibited significant characteristics of MASLD 30 days postpartum. GDM dams developed MASLD and MASH with progressive steatosis, ballooning, inflammation, and mild postpartum fibrosis compared to HF and LF dams. Additionally, GDM dams showed excessive lipid storage and increased cell turnover in liver sections associated with increased mitosis, apoptosis, and anisokaryosis. To study MASH in the context of diabetes, the combination of a HF diet with a low dose of streptozotocin has been used before in male mice^51^. Our studies differ as we are using a shorter period of HF diet feeding in female mice and include pregnancy as an additional metabolic stress factor. Earlier examination of our GDM model showed no differences in hepatic triglycerides at the end of the gestation period at GD18. However, by PP15, there were some indications of the early development of MASLD in the GDM dams, including mildly elevated MASLD activity scores and increased hepatic triglyceride levels^19^. Plasma AST and ALT levels were, however, only increased in GDM dams 30 days postpartum. This data combined shows a progressive development of MASLD postpartum in our GDM model.

Dams subjected to an extended HF diet exhibited increased body weights and gonadal adipose pad weights. Surprisingly, despite the HF feeding, GDM dams consistently displayed a lean phenotype postpartum. The limited beta-cell capacity resulting in decreased insulin levels in the GDM dams could play a role in the protection against HF diet-induced weight gain, as hyperinsulinemia has been implicated as causal factor to diet-induced obesity^52^. Although dietary recommendations are provided to women with GDM in standard care^53^ and have been shown to be effective^54–56^, lifestyle intervention programs may not work to reduce long-term risks such as MASLD in lean insulin secretion-driven GDM. Consequently, there is a need for interventions aimed at improving maternal glucose levels in late pregnancy and/or the postpartum period to mitigate the development of MASLD for specific GDM subtypes.

In conclusion, we show MASLD development in our lean GDM model postpartum. The results of our study show that pregnancy hyperglycaemia driven by impaired insulin secretion in absence of obesity has a clear contribution to the development of postpartum MASLD. Further research efforts will be dedicated to elucidating the precise mechanisms underlying the development of MASLD in insulin-secretion-driven GDM. Understanding of these intricacies, leads to valuable insights to develop targeted interventions that benefit lean GDM patients and help to enhance our understanding of the complex interplay between metabolic factors and liver health in the context of pregnancy-related hyperglycaemia.

### Supplementary Information

Supplementary Information.

## Data Availability

All data supporting the findings of this study are available within the paper and its Supplementary Information.

## Abbreviations

ALT: Alanine aminotransferase
AST: Plasma aspartate transaminase
BG: Blood glucose
NEFA: Non-esterified fatty acids
GD: Gestational day
GDM: Gestational diabetes mellitus
gWAT: Gonadal white adipose tissue
HF: High fat diet
HFSTZ: High fat diet + Streptozotocin
LF: Low Fat diet
NA: Non-applicable
MASLD: Metabolic dysfunction-associated steatotic liver disease
MAS: MASLD activity score
OGTT: Oral glucose tolerance test
PDM: Pre-gestational diabetes mellitus
PP: Postpartum day
pWAT: Perirenal white adipose tissue
RBG: Random blood glucose
STZ: Streptozotocin
sWAT: Subcutaneous white adipose tissue
TG: Triglycerides
T2DM: Type 2 diabetes mellitus
