# Supplementary material for: Postpartum development of metabolic dysfunction-associated steatotic liver disease in a lean mouse model of gestational diabetes mellitus
Source: Sci Rep. 2024 Jun 25;14:14621. doi: 10.1038/s41598-024-65239-2 (PMC11199516; doi:10.1038/s41598-024-65239-2)
Supplement: Supplementary file 1 — Supplementary Information. [file 41598_2024_65239_MOESM1_ESM.pdf]

Supplementary materials

Sample size calculation

The sample size was calculated using G*Power (http://www. gpower. hhu. de/), with β=0.9, α=0.05, effect size=1.67 (mean difference=5, SD=3), and identical group sizes. The effect size was based on the previously observed difference in blood glucose levels between GDM and LF dams at 20 min (peak glucose in LF control dams) during the OGTT (Li et al., 2020). This resulted in a mean group size of 9. Due to the expected dropout related to the development of PDM, pregnancy rate, and lost litter, the final animal numbers are different. In total, 38/74 (45%) mice were excluded from this study. This resulted in 13 GDM, 12 HF, and LF 10 dams. The final numbers are shown in **Supplemental Table 1.** The number of breeding males required was determined assuming that each day, a maximum of 1 in 3 dams would be in proestrus.

Supplemental Table 1: Number of female breeders, number of pregnant mice, lost litters, exclusions, and final number of animals included in the analyses.

Lost litter = death of all pups and/or cannibalization between birth and PP2. * Dams developed pre-gestational hyperglycaemia (RBG> 12 mmol/L) and were excluded from the study prior to breeding. †: The human endpoint was reached because of excessive weight loss (>15% compared to baseline) prior to breeding. LF, low-fat diet; HF, high-fat diet; GDM, gestational diabetes mellitus; PDM, pre-gestational diabetes mellitus; GD, gestational day; PP, postpartum day; NA, not applicable.

	Female mice	Mated	Pregnant (%)	Lost litter (%)	Excluded	Reason exclusion	Included till PP30
Total	74						35
GDM	40	21	14 (70%)	1 (7%)	19	19 PDM *	13
HF	17	17	14 (82%)	2 (14%)	NA	NA	12
LF	17	17	12(75%)	3 (25%)	1	1†	10

Supplemental Table 2: Random glucose and insulin time points

Data are presented as the mean ± SD. Statistical analysis was performed using Kruskal-Wallis test, followed by Dunn's multiple comparison test. P values are presented as group comparisons. LF, low-fat diet; HF, high-fat diet; GDM, gestational diabetes mellitus; GD, gestational day; PP, postpartum day.

	**LF** N=10	**HF** N=12	**GDM** N=13	LF vs. HF	LF vs. GDM	HF vs. GDM
**Random blood glucose (mM)**						
**GD -14**	7.09 ± 0.55	7.55 ± 0.82	7.68 ± 0.61	ns	ns	ns
**GD0**	8.01 ± 1.12	7.38 ± 0.62	9.51 ± 1.49	ns	0.036	0.0006
**GD7**	8.18 ± 0.64	6.58 ± 3.12	10.38 ± 1.47	ns	0.0004	0.0042
**GD14**	7.89 ± 0.66	7.9 ± 0.9	10.54 ± 2.03	ns	0.0022	0.0024
**GD18**	7.82 ± 0.51	7.38 ± 0.86	14.8 ± 4.53	ns	0.0003	0.0002
**PP2**	7.41 ± 0.96	6.67 ± 0.75	10.66 ± 3.42	ns	0.0146	0.0032
**PP8**	7.23 ± 0.69	6.81 ± 0.81	7.88 ± 2.19	ns	ns	ns

**PP15**	7.69 ± 0.87	6.83 ± 0.84	8.55 ± 2.77	ns	ns	ns
**PP23**	7.71 ± 0.91	7.87 ± 0.73	11.86 ± 2.7	ns	0.0003	0.0004
**PP30**	8.77 ± 0.86	8.94 ± 0.79	12.98 ± 3.52	ns	0.0027	0.0037
**Random blood Insulin (mU/L)**						
**GD0**	33.51 ± 16.99	44.37 ± 17.25	25.24 ± 7.47	ns	ns	0.0099
**GD18**	43.06 ± 23.05	23.00 ± 5.76	26.28 ± 5.18	0.0398	ns	ns
**PP15**	24.33 ± 8.54	18.30 ± 5.76	26.34 ± 15.65	ns	ns	ns
**PP30**	20.36 ± 9.45	23.04 ± 11.61	21.32 ± 4. 94	ns	ns	ns

Supplementary Table 3: GD15 and PP28 OGTT glucose, OGTT insulin, HOMA IR and Matsuda Index

Data are presented as the mean ± SD. Statistical analysis was performed using Kruskal-Wallis test, followed by Dunn's multiple comparison test. P values are presented as group comparisons. LF, low-fat diet; HF, high-fat diet; GDM, gestational diabetes mellitus; GD, gestational day; PP, postpartum day.

	**LF** N=10	**HF** N=12	**GDM** N=13	**LF vs. HF**	**LF vs. GDM**	**HF vs. GDM**
**GD15**						
**HOMA IR**	9.68 ± 1.13	10.43 ± 2.29	13.69 ± 2.32	ns	0.0016	0.0109
**MATSUDA Index**	2.47 ± 0.22	2.25 ± 0.43	1.32 ± 0.20	ns	<0.0001	0.001
**OGTT Glucose clearance (mM)**						
**t0**	7.04 ± 0.50	7.54 ± 0.45	9.97 ± 1.72	ns	0.0001	0.0007
**t5**	10.19 ± 1.82	11.51 ± 1.33	15 ± 3.78	ns	0.0022	0.0178
**t10**	13.21 ± 2.49	14.25 ± 1.99	18.73 ± 4.02	ns	0.0017	0.0059
**t20**	11.14 ± 1.32	14.83 ± 2.58	24.36 ± 3.87	0.0013	<0.0001	<0.0001
**t30**	9.77 ± 1.16	11.63 ± 2.78	25.72 ± 2.56	ns	<0.0001	<0.0001
**t45**	9.44 ± 0.76	10.11 ± 1.27	24.61 ± 2.24	ns	<0.0001	<0.0001
**t60**	9.2 ± 0.49	9.97 ± 1.02	23.69 ± 2.27	ns	<0.0001	<0.0001
**t90**	7.53 ± 0.55	7.78 ± 0.9	21.28 ± 1.92	ns	<0.0001	<0.0001
**t120**	7.21 ± 0.54	7.1 ± 0.86	19.62 ± 2.21	ns	<0.0001	<0.0001
**OGTT Insulin release (mU/L)**						
**t0**	34.99 ± 4.3	35.78 ± 7.05	36.7 ± 3.42	ns	ns	ns
**t5**	42.08 ± 7.16	49.83 ±16.72	39.62 ± 7.28	ns	ns	ns
**t10**	49.68 ±10.47	53.1 ± 11.64	42.38 ± 8.34	ns	ns	ns
**t30**	42.8 ± 9.19	45.05 ± 8.03	44.04 ± 7.01	ns	ns	ns
**t60**	43.46 ± 7.74	44.1 ± 6.14	42.03 ± 5.83	ns	ns	ns
**t120**	41.84 ± 8.37	42.95 ± 8.56	44.34 ± 5.10	ns	ns	ns
**PP28**						
**HOMA IR**	3.85 ± 3.19	6.42 ± 3.55	5.32 ± 2.69	ns	ns	ns
**MATSUDA Index**	8.17 ± 3.20	5.11 ± 2.82	5.71 ± 1.57	ns	ns	ns
**OGTT Glucose clearance (mM)**						
**t0**	7.41 ± 0.6	8.66 ± 1.29	12.86 ± 2.58	0.0218	<0.0001	0.0002
**t5**	10.66 ± 2.06	13.07 ± 1.88	17.46 ± 3.87	0.0274	<0.0001	0.005
**t10**	12.88 ± 2.57	18.64 ± 2.37	23.04 ± 3.43	<0.0001	<0.0001	0.0031
**t20**	11.89 ± 1.67	15.39 ± 1.34	26.82 ± 2.55	0.0001	<0.0001	<0.0001
**t30**	10.69 ± 0.9	11.73 ± 0.93	26.45 ± 2.55	0.0382	<0.0001	<0.0001
**t45**	9.98 ± 0.76	12.09 ± 1.5	24.79 ± 2.74	0.0015	<0.0001	<0.0001
**t60**	9.99 ± 1.19	12.19 ± 1.71	24.61 ± 2.99	0.0057	<0.0001	<0.0001

**t90**	8.17 ± 1.02	11.27 ± 1.15	20.68 ± 2.35	<0.0001	<0.0001	<0.0001
**t120**	8.49 ± 0.79	10.08 ± 0.99	19.27 ± 2.95	0.0012	<0.0001	<0.0001
**OGTT Insulin release (mU/L)**						
**t0**	6.4 ± 0.79	20.51 ±21.38	7.92 ± 4.32	ns	ns	ns
**t5**	20.19 ±14.84	51.39 ±34.13	11.98 ± 8.8	ns	ns	0.0323
**t10**	29.16 ±16.87	97.75 ±24.94	11.98 ± 7.13	ns	<0.0000	<0.0001
**t30**	17.38 ± 5.42	26.9 ± 14.58	10.57 ± 5.35	ns	ns	0.0383
**t120**	29.48 ±12.65	30.89 ±14.87	11.55 ± 3.36	ns	0.021	0.0184

Supplementary Table 4. PP30 liver weight, lipid content and morphology. Data are presented as the mean ± SD. Statistical analysis was performed using Kruskal-Wallis test, followed by Dunn's multiple comparison test. P values are presented as group comparisons. LF, low-fat diet; HF, high-fat diet; GDM, gestational diabetes mellitus; GD, gestational day; PP, postpartum day.

	**LF** N=10	**HF** N=12	**GDM** N=13	**LF vs. HF**	**LF vs. GDM**	**HF vs. GDM**
**Total Cholesterol (µmol/g)**	14.92 ± 3.05	8.94 ± 0.96	12.03 ± 1.49	<0.0001	ns	0.0038
**Free Cholesterol (µmol/g)**	5.86 ± 0.60	5.90 ± 0.69	5.80 ± 0.80	ns	ns	ns
**Cholesterol-esters (µmol/g)**	9.06 ± 2.74	3.04 ± 0.79	6.23 ± 1.07	<0.0001	ns	0.0024
**TG (µmol/g)**	33.03 ± 7.90	33.58± 11.36	65.92 ± 9.33	ns	0.0002	0.0002
**PL (µmol/g)**	32.11 ± 3.01	36.53 ± 4.97	34.82 ± 5.22	0.0454	ns	ns
**TG/PL**	1.03 ± 0.23	0.96 ± 0.46	1.91 ± 0.29	ns	0.0032	<0.0001
**Glycogen (mg/g)**	68.86 ± 15.00	53.77 ± 8.43	52.26 ± 9.05	ns	0.0321	ns
**Steatosis Grade**	0.65 ± 0.71	0.17 ± 0.44	1.50 ± 0.84	ns	ns	0.0004
**Ballooning Grade**	0.00 ± 0.00	0.00 ± 0.00	0.85 ± 0.63	ns	0.0002	0.0002
**Lobular inflammation Grade**	0.80 ± 0.42	0.83 ± 0.39	1.08 ± 0.28	ns	ns	ns
**MASLD (MAS) Score**	1.45 ± 0.80	1.00 ± 0.64	3.42 ± 1.12	ns	0.0164	<0.0001
**Apoptotic cell (per 3000 hepatocytes)**	0.11 ± 0.18	0.06 ± 0.14	2.49 ± 1.76	ns	0.0022	0.0002
**Mitoses (per 3000 hepatocytes)**	0.00 ± 0.00	0.03 ± 0.10	3.36 ± 3.56	ns	0.0025	0.0048
**Anisokaryosis**	1.30 ± 0.35	0.42 ± 0.51	2.77 ± 0.33	ns	0.0079	<0.0001
